# Optimizing patient engagement to enhance a learning health system

**DOI:** 10.3389/frhs.2025.1607662

**Published:** 2025-10-13

**Authors:** Mikie Mork, Allison Strilchuk, Jatin N. Patel, Donna Smith, Gloria Wilkinson, Adam Brown, Seija Kromm, Michele Dyson, Tracy Wasylak

**Affiliations:** ^1^ Formerly Alberta Health Services, Strategic Clinical Networks and Provincial Programs, Calgary, AB, Canada; ^2^ Cumming School of Medicine, University of Calgary, Calgary, AB, Canada; ^3^ Formerly Alberta Health Services, Engagement and Patient Experience, Calgary, AB, Canada; ^4^ Former SCN Patient and Family Advisors and Co-chairs of the Patient Engagement Reference Group, Calgary, AB, Canada; ^5^ Partnerships and Innovation, Acute Care Alberta, Calgary, AB, Canada; ^6^ Department of Community Health Sciences, Cumming School of Medicine, University of Calgary, Calgary, AB, Canada; ^7^ Applied Research and Innovation, Recovery Alberta, Edmonton, AB, Canada; ^8^ Department of Psychiatry, Faculty of Medicine and Dentistry, University of Alberta, Edmonton, AB, Canada; ^9^ Faculty of Nursing, University of Calgary, Calgary, AB, Canada

**Keywords:** patient engagement, patient-oriented research, patient engagement indicators, learning health system, health services research, clinical networks

## Abstract

**Background:**

Patient and family advisors have served as an integral part of a collaborative, province-wide learning health system in Alberta for more than a decade, contributing to evidence generation, knowledge mobilization and research activities focused on improving patient outcomes.

**Objective:**

This paper describes how Alberta Health Services (AHS) and the Strategic Clinical Networks™ (SCNs™) (i) embedded patient engagement and patient-oriented research in health services innovation and improvement, including project planning, co-design, execution and decision-making, (ii) created opportunities for patient advisors to participate in leadership committees, research panels and keynote addresses, (iii) co-designed engagement practices, resources and supports with patients and community partners, and (iv) applied a mixed-methods approach for assessing engagement effectiveness.

**Methods:**

AHS patient advisors collaborated with provincial partners and researchers, including the Alberta Strategy for Patient-Oriented Research (SPOR) Support Unit (AbSPORU) Patient Engagement Team, to co-design and pilot a standardized set of patient and family engagement indicators (PFE-Is) that could be used to evaluate engagement effectiveness and improve current practices. Through surveys and consultations with key interest holders, the team established a baseline for effective engagement and built consensus for patient engagement priorities, recommendations, and actions to improve patient and family engagement.

**Results:**

Five themes emerged from consultations with advisors and AHS staff: supports for engagement, learning together, diversity of perspectives, the role of advisors, and evaluating meaningful patient engagement. Recommendations and actions to strengthen patient engagement emerged that build on existing practices and supports, and include opportunities to improve resources, foster inclusivity, and promote collaborative learning opportunities.

**Discussion:**

The evidence-based PFE-Is and survey are ready for implementation across Alberta's health system to monitor and evaluate patient and family engagement, gather feedback from advisors and staff, and refine current strategies and practices. Continued collaboration with patient and family advisors is expected to support progress as a learning health system and strengthen the ability of provincial health agencies to generate actionable insights, drive improvements, and deliver high quality, patient-centred care.

## Introduction

Learning health systems (LHSs) use data, evidence, and knowledge to drive systematic improvement and innovation. They rely on measurement, evaluation, and engagement to transform data and knowledge into insights that inform clinical practice, service delivery, and health policy. While many health systems refer to an LHS approach, there remains considerable variation in what this means in terms of processes, roles, structures and frameworks (1–3). The role of patients and families in an LHS is an evolving area of research, including specific ways in which patient engagement and patient-oriented research are embedded to advance the goals of an LHS; their roles and contributions; and the extent to which they influence clinical practice and outcomes.

The literature includes many definitions for what constitutes an LHS. Some models emphasize *data and analytics* and focus primarily on analytic structures and systematic workflows to gather and apply evidence, monitor and improve outcomes, and iterate (1). Other models emphasize *cycles of rapid improvement that occur at every level of the health system*, creating an environment where timely production, use, and sharing of evidence and data exists, as well as rapid evaluation, feedback, and adaptation (2, 3). Propelling these rapid improvement cycles are *multiple forms of evidence* that deliver insights and support decision making. These include health data as well as qualitative insights, jurisdictional scans, and key informant interviews.

A growing body of evidence suggests patient engagement can support improvements in quality of care, health outcomes, and the experience of patients and healthcare providers (4, 5). However, strategies, structures, and processes to enable patient engagement and patient-oriented research vary widely, as do methods of gauging their effectiveness and impact.

This paper describes ways in which Alberta Health Services (AHS), Canada's first fully integrated provincial health delivery organization, embedded and engaged patient and family advisors as part of an LHS, and specifically as part of Alberta's Strategic Clinical Networks™ (SCNs™). It outlines approaches for incorporating patient input at leadership and decision tables, and as part of project planning, co-design and execution. Further, it shares examples of ways patient advisors supported health services research, improvements and innovation across the care continuum, and AHS' approach for monitoring and assessing engagement effectiveness. These approaches are adaptable to other health contexts and jurisdictions interested in advancing an LHS.

## Patient engagement within a learning health system

LHSs vary in the extent to which they embed bottom-up (patient, clinician and community-led) approaches and co-design (2), and their attention to (and investment in) building competencies for rapid learning and improvement—including data literacy, implementation and decision supports at varying levels of the health system (1, 2).

The LHS Action Framework developed by Reid et al. emphasizes the importance of integrating patient partners into LHS processes, and connecting partners and assets using a broad system lens. The authors state that “*Central to the LHS…is the co-design of care approaches that are informed by local data and high-quality evidence syntheses, tailored to local context, and aligned to address foreseeable barriers to adoption and maintenance. This requires direct engagement with people who are impacted by the problem at hand – patients, caregivers, care providers, community members – along with those who have the ability to influence or are directly involved in the co-designed service – healthcare professionals, managers, and health system operators*” (3).

Alberta is fortunate to have many assets that support an LHS. Strategic clinical networks (SCNs) have helped support an LHS that is anchored on patients' needs, perspectives and aspirations; generate and mobilize data and evidence; and align strategic directions with organizational priorities. From 2012 to 2024, SCNs worked provincially to advance care, improve outcomes and drive innovation in specific areas of health (e.g., bone and joint health; cancer; cardiovascular health and stroke; critical care; maternal-child health; etc.). These structures, together with Provincial Programs in addiction and mental health, Indigenous health, primary care, population and public health, and seniors health, served as engines of innovation and improvement, where health system leaders, clinicians, researchers, patient advisors and community partners could collaborate to develop sustainable solutions to complex health challenges, evaluate new models of care, and advance practices that improve quality, outcomes and value (6).

Another key asset has been the province-wide implementation of an integrated clinical information system (Epic®) that enables evidence-gathering, measurement, and evaluation by frontline clinicians. Epic (Connect Care) provides a powerful tool that supports data capture and analysis provincially, accelerating progress for learning and improvement. Implementation was completed in 2024, and the system is operational at 800+ AHS sites (including hospitals, continuing care centres, community ambulatory care, pharmacy, diagnostic imaging, laboratory services, and affiliate sites). Work is currently underway to optimize this system and build reporting dashboards and processes that support real-time data extraction and decision making.

Learning health systems can take many forms (e.g., centralized models, decentralized health regions or zones, independent hospital boards), all of which benefit from clear objectives, accountability, and effective partnerships. Although the structures that facilitate patient and family engagement may vary, any system that engages patients and families would benefit from reliable, evidence-based indicators of engagement effectiveness, and ongoing evaluation of engagement supports, activities, and the experience of patient and family advisors and healthcare staff. As such, we are confident that the study findings are applicable to health systems with different structures and contexts.

Sponsorship and support from executive leadership is critical to successful patient engagement. In the absence of SCN-like structures, patient engagement can be implemented at a system-wide or local level (or both) with appropriate leadership support, processes, policies, resources, and guidelines. An example is the former Calgary Health Region (which pre-dated AHS and the SCNs), where Quality Assurance Structures included patient and family advisors.

## Patient engagement and patient-oriented research within clinical networks

Over the past decade, AHS has continued to refine its strategies for patient engagement, focusing on improved communication, team-based approaches, and embedding patient advisors and patient-oriented research as an integral part of its operations and efforts to improve patient outcomes, service delivery, and performance (6).

Every SCN and Provincial Program included embedded patient and family advisors and operated with an understanding that patients and families must be actively involved in setting priorities and co-designing solutions. The “nothing about us without us” approach was a guiding principle for the SCNs since their inception. They brought together diverse partners to co-design the strategies and solutions needed to address priorities such as unwarranted variation in care, excessive wait times, and barriers to care (7). All networks used data and evidence to identify gaps and leveraged the unique knowledge, skills, lived experience and perspectives of all partners. Though not responsible for health policy or decision-making, SCNs provided evidence-informed guidance to government and executive health leaders, rigorously evaluated and refined practice changes in clinical settings, and supported the implementation and mobilization of evidence into practice to support system change, improvement and innovation (8).

In the twelve years SCNs operated, approximately 150 patient and family advisors contributed every year to their work and its impact for the people of Alberta. All advisors were volunteers and filled various roles, serving as committee and working group members, project co-leads, and patient and community engagement researchers (PaCERs). In addition, approximately 1,000 citizens volunteered as patient and family advisors on Alberta's health advisory councils, provincial advisory councils, and patient and family advisory committees (PFACs) (9).

### Recruitment

SCN Patient and Family Advisors were recruited in several ways. Albertans who were interested in volunteering with AHS would contact AHS' Volunteer Services, who would refer them to the SCNs if they were of interest. Clinicians were also able to recruit volunteers who were patients and/or family members in their clinics.

PFAC was overseen by the Engagement and Patient Experience department and was a separate entity from the SCNs. Any AHS advisor (regardless of where they volunteered) could apply to become an advisor with PFAC; however, there was limited overlap between PFAC and SCN advisors (of the approximately 28 PFAC advisors, two were also SCN advisors). While these were highly motivated patient volunteers, most advisors focused their efforts in specific areas of interest based on their lived experience.

### Diversity of perspectives

Seeking diverse perspectives is a known challenge for patient engagement. Among the important patient engagement principles we consistently heard from SCN patient and family advisors was that they could and would only speak for themselves and to their individual lived experience(s)—not for others. We recognize that advisors provide input based on that experience and the type of care services they have received (inclusive of processes and policy). SCNs were fortunate to have advisors whose experience spanned many health disciplines and sectors, including specialty areas of care (cancer, cardiology, etc.). AHS does not collect demographic information from its advisors, so many invisible (deep-level) attributes were not known (e.g., religious beliefs, neurodiversity, family status, cultural background, socioeconomic status, gender identity, values and beliefs).

Research with marginalized/vulnerable groups relies heavily on self-identification and is regulated by government policy (10). Although the SCN patient and family advisors invited to share their feedback reflected diversity in their interactions and lived experience with the health system (e.g., experiences with primary care, acute care, specialist care (cancer, cardiology, etc.), and treatment across many disease areas and healthcare settings (urban, rural and remote), SCN leadership and its Patient and Family Engagement (PFE) team recognized that advisors who identify as belonging to vulnerable, marginalized or racialized groups were underrepresented in our SCN patient and family advisor population. The same underrepresentation was recognized across Alberta Health Services, with health equity data increasingly demonstrating that people belonging to vulnerable, marginalized or racialized communities disproportionally experience adverse health outcomes (11).

Recognizing the value of prioritizing diversity and inclusivity, the SCN PFE team recruited a student from the University of Alberta's Experiential Learning in Innovation, Technology, and Entrepreneurship (ELITE) program for Black Youth in 2022 to compile information that would support the development of an SCN Patient and Family Advisor Diversity Engagement Strategy. Through this work, the team aimed to better understand (i) barriers that hinder these populations from contributing to the work of the SCNs, (ii) best approaches for bringing these voices to SCN planning tables, and (iii) needs of patient and family advisors who belong to vulnerable, marginalized or racialized populations. The intent was to develop strategies SCNs could use that would facilitate wider engagement of persons from vulnerable, marginalized and racialized groups to bring their perspectives to the work of SCNs in ways that were appropriate and supportive to these populations.

Unfortunately, work on this strategy appears to have paused (at least temporarily) during the health system restructuring and stand-down of SCNs. There has been no announcement or further update on the continued development, or implementation, of a Patient and Family Advisor Diversity Engagement Strategy within AHS or the four new provincial health agencies, although we hope this important work will resume once staff transitions associated with the health system restructuring are complete, and there is greater capacity across the system.

## Evolving practices, resources and supports

As part of their 2019–2024 strategic plan, SCN leaders identified patient engagement as an area of focus, committing to “strengthening relationships with patient and family advisors, engaging them as equal partners in decision-making, and prioritizing work that improves health outcomes and patient and family experiences” (12). This objective was reinforced by key interest holders during consultations and approved and endorsed by AHS executive leaders, providing the leadership support needed to advance these aims.

As networks grew in experience, they continued to refine processes, resources and supports for patient and family advisors based on learnings and feedback from advisors, network partners, and health system leaders. This included expanding opportunities for advisors to contribute to the networks in leadership roles, with flexibility around role definition.

Many advisors chose to volunteer with a specific SCN, reflecting an area of interest and/or lived experience. Advisors would co-lead projects, co-design solutions, and collaborate as integral team members on priority initiatives and health services research (13). Other advisors supported multiple networks or activities, some in a shared capacity, collaborating with other advisors on PFACs. Patients, families and caregivers also contributed their voices and input as members of committees or working groups, participating in direction setting, prioritization, planning, implementation, and knowledge mobilization; or as Patient and Community Engagement Researchers (PaCERs).

PaCERs are people with lived experience who receive training in qualitative health research through the University of Calgary, Department of Continuing Education (Calgary, Alberta, Canada), in partnership with the Alberta Strategy for Patient-Oriented Research (SPOR) Support Unit (AbSPORU), a provincial partner in the health innovation, improvement and research space. PaCERs work with clinical researchers, health systems and community organizations, conducting research or quality improvement studies that support priority areas for patients and for the health system. The PaCER program is unique to Alberta and reflects a new approach to patient engagement in health research, aiming to transform the role of patients and community in health system research, practice, planning and policy and “create a patient-informed research voice” (14). More than 100 community members with lived experience have gone through PaCER training (15), and their contributions to an LHS are evident in many areas of health. Examples include building research capacity in Indigenous communities and understanding the patient experience of adolescents with concussions, patients navigating bladder cancer, and those living with chronic pain (14, 16). For information on specific patient-oriented research projects and their impact, including how PaCERs “bring patient-informed research evidence into health care planning, policy and practice”, see https://www.ucalgary.ca/patient-community-engagement-research.

These opportunities for patient engagement and patient-oriented research are consistent with Reid et al.'s LHS framework in which patient, caregiver and provider co-design is a central component and involvement of patient and family partners, community partners, and Indigenous and other equity deserving groups (as defined by Reid et al.) are accelerators for an LHS (3).

Engagement to support development of the SCN Roadmap reaffirmed the importance of continuing to ensure adequate supports for patient and family engagement. A dedicated SCN Patient and Family Engagement Team provided leadership, administrative, communication and logistics support for advisors. The team included staff liaisons who served as a direct point-of-contact for advisors. They also provided a forum known as the Patient Engagement Reference Group (PERG) for advisors to regularly meet (in person and virtually), share experiences and learn from one another. Feedback from this group strongly indicates that advisors valued these peer-to-peer learning experiences, and the opportunity for advisors to build connections, expand their knowledge of current activities, and derive inspiration from one another (8).

Among the supports developed by the SCN Patient and Family Engagement Team was a Patient Engagement Orientation that brought staff liaisons and advisors together to learn about their roles and build connections. Orientations were provided three times a year, co-hosted by an advisor and staff liaison, and attended by new and existing advisors and staff. Attendance was optional; however, SCNs recommended all staff and advisors participate in at least one orientation as part of onboarding to build a shared understanding of engagement principles (16).

A key learning for the SCNs was the need for continuous dialogue, evaluation, and refinement of supports and resources with patient and family advisors and PaCERs. Among the resources co-designed by advisors and SCNs to support effective patient engagement across all networks were “Engaging for Excellence” guides – one for SCN staff liaisons and another for advisors (16). These guides shared best practices for patient engagement, role descriptions for advisors and liaisons, and provided guidance on expense reimbursement. They also shared guiding principles for meaningful engagement, including fostering and maintaining mutual respect, type and timing of engagement (e.g., flexible meeting times, including evenings), co-design principles, and techniques to level the playing field (e.g., avoid titles and jargon) and support effective communication.

## Assessing the impact and value of advisor contributions in advancing a learning health system

Allen et al. identifies patient and family engagement as an output measure when operationalizing and evaluating impact in an LHS and recommends that “*In addition to measuring…the total number of patients included as partners, organizations should document how recommendations from patient and family stakeholders are applied so stakeholders know the value of their input*” (17). A systematic review by Bombard et al. further found that following up with advisors and communicating how their contributions added value to the work is important to sustain their interest and engagement (5).

To this end, in 2019, the SCNs began highlighting PaCER research as part of its annual impact reporting, and expanded its reporting further in 2021 to include a patient engagement summary. The summary is largely qualitative, listing examples of advisor, PFAC and PaCER contributions over the past year, key deliverables and other outputs. These reports also include the voice of SCN patient and family advisors about their experience and the value of the work (13).

The 2023–24 report includes several examples of co-designed patient-facing resources (e.g., a hospital to home transition guide, prehabilitation information on the MyHealth. Alberta public-facing website, and patient journey maps for patients visiting emergency departments and those undergoing acute kidney dialysis) (13). In 2023–24, advisors also conducted patient interviews for quality improvement work involving enhanced lipid reporting (ELR), and another served as a co-investigator on a research study focused on pressure injury prevention, helping inform decisions about study design, data collection and analysis.

These examples highlight just a few of the many contributions patient advisors have made as embedded, valued partners within the SCNs. Their involvement spanned all stages of health innovation, including early research and pilot studies, prioritization and implementation and mobilization of new knowledge into practice. While it is difficult to assess the impact of advisor contributions from those of the initiative as a whole, the examples clearly demonstrate that patient advisors helped advance improvements in care, created resources to improve experience of patients and families, helped prioritize actions, co-design solutions, and co-lead work that matters to patients, families, and community members.

## Measuring and evaluating patient engagement and its effectiveness

Reliable, evidence-based indicators are essential to an LHS, enabling it to monitor quality, assess progress over time, and identify potential areas for improvement. Defining and implementing appropriate, evidence-based indicators for patient and family engagement, and establishing feedback cycles of learning and improvement, enables health systems to evaluate and improve their engagement processes, practices and supports.

In addition to tracking the number of active patient and family advisors, SCNs began monitoring advisor retention rates using a volunteer database and conducting an annual patient engagement survey to gather feedback on what was going well and identify areas of improvement. SCNs used this data to refine strategies for patient engagement and improve advisor satisfaction, utilization, and retention (18). Some networks also conducted one-on-one interviews with advisors to gather additional information and feedback.

Advisors directly influenced the development and refinement of indicators of engagement effectiveness, and their implementation across the SCNs. As part of a collaborative, patient-oriented research project that began in 2021, AHS patient and family advisors, researchers from the University of Calgary (Dr. Maria Santana, Dr. Paul Fairie, and Dr. Tamara McCarron), the AbSPORU Patient Engagement Team (Sadia Ahmed, Sandra Zelinsky), and the SCNs partnered on a mixed-methods study to identify a set of patient engagement indicators that could be used provincially to measure patient and family engagement and the experience of advisors and staff working together. The process of co-developing and validating these indicators is described in a joint publication by Santana et al. (19).

The indicators recommended by Santana et al. reflected seven themes: communication (e.g., having enough information to be able to carry out one's role as an advisor); comfort in contributing (e.g., ability to express views freely); supports for effective engagement; impact and influence of the engagement; diversity of perspectives; respectful engagement; and working together (e.g., project design, execution, disseminating results). Through this process, eighteen indicators were accepted and recommended for implementation.

This work provides a tangible example of co-design and an exemplar to further understand how patient and family engagement indicators (PFE-I) may be used as a measurement tool within an LHS. As Santana et al. describes “*the participatory approach used to develop PFE-Is…ensures that engagement was evaluated from the perspective of those who provide and receive care*…*These newly developed indicators present an opportunity to improve meaningful engagement ensuring that the voices of the individuals with lived experiences are incorporated into health systems supporting the transformation of healthcare”* (19).

Of note, the indicators defined by Santana et al. focused on the *engagement experience* (advisor- and staff-reported measures) and opportunities to strengthen engagement processes and supports. The study did not include standardized indicators to assess the *impact* of engagement efforts on other outcomes of interest to an LHS (e.g., health outcomes, policy, health system performance, or the experience of patients and providers in receiving or delivering care).

## Methods

This mixed-methods, multiphase project builds on the work jointly conducted with Santana et al. and evaluates the feasibility of implementing these indicators to measure patient and family engagement and the experience of advisors and staff working together. As described in Santana et al., existing measures of patient and family engagement were identified through a review of the literature and consultations with patients, caregivers, community members and researchers (19). The Public and Patient Engagement Evaluation Tool (PPEET) (20), a tool developed by researchers and public and patient engagement practitioners, was selected.

The next phase of study included a survey and one-on-one semi-structured interviews, which aimed to further explore the use of PPEET in this context. Survey results and interviews informed the development of PFE-Is, and the team used an evidence-based Delphi consensus process (using a modified RAND/UCLA Appropriateness Method) to identify and refine a core set of PFE-Is (19).

### Online survey and consultations

An online survey was emailed to 293 individuals (175 advisors, 69 SCN staff members and 49 SCN leaders) in spring 2023. Selection bias was minimized by including all active SCN patient and family advisors, SCN leaders and core SCN staff (which included Senior Provincial Directors, Senior Medical Directors, Senior Provincial Officers, Scientific Directors, Assistant Scientific Directors, Executive Directors, Managers, Staff Liaisons, and Senior Consultants for all SCNs).

We received 96 completed surveys (32.8% response rate). This included surveys from 51 advisors, 31 SCN staff, and 14 SCN leaders. PFE-Is were then drafted based on the questions from the PPEET survey. Patient and family advisors and select AHS staff were invited to complete the survey about their engagement experiences. The AbSPORU Patient Engagement team led the survey deployment, data analysis, and reporting (19).

Following the survey, a Patient Engagement Working Group was created, bringing AHS staff and advisors together to review the survey results, advise on consultations, and co-develop recommendations to improve meaningful engagement. From November 2023 to February 2024, six consultations and three drop-in sessions were held to gather feedback from staff and advisors. Standardized questions were developed, and the Working Group Chair facilitated the consultations with support from Working Group members. Everyone who received a survey invite was invited to participate in a consultation, again to minimize selection bias.

### Consensus process and recommendations

Following the consultations, the Patient Engagement Working Group qualitatively analyzed the consultation data, identifying themes and key messages. Recommendations for patient engagement improvement were drafted, and consensus was achieved by group discussion and verbal agreement on final recommendations. The Working Group used a participatory approach to identify proposed actions for each of the recommendations.

### Barriers and limitations

The barriers that were encountered are common in research studies. These included time constraints (both for researchers and participants), limited funding, resources, and sample size/response rate.

## Results

Five themes emerged from consultations with advisors and AHS staff (Table 1): supports for engagement, learning together, diversity of perspectives, exploring the role of advisors, and evaluating meaningful patient engagement. These themes provide evidence of important considerations when engaging patient advisors as part of an LHS.

**Table 1:** Themes and key messages from consultations on patient and family engagement (2023–2024).

Theme	Key messages
Supports for engagement	• Effective engagement requires early involvement of advisors in planning stages, inclusion in decision-making from the start (design phase), and clear role definitions for advisors and staff seeking engagement. • Having a designated staff liaison to support advisors is highly valued and seen as a critical enabler for successful engagement.
Learning together	• Opportunities exist to improve and expand knowledge resources (written, peer-to-peer and collaborative learning) that support patient engagement, continue to refine processes and share new engagement practices.
Diversity of perspectives	• Participants expressed desire to broaden engagement of advisors to reflect new and existing populations in Alberta. • Feedback revealed need to clarify understanding about what equity and diversity means within advisory roles.
Exploring the role of advisors	• Advisors bring unique perspectives and lived experience to the table. They seek more active participation, desiring to co-facilitate meetings to introduce diverse viewpoints, particularly their lived experience. • Challenges exist with the voluntary nature of the advisory role (e.g., balancing time and contributions, tokenism vs. meaningful engagement). • A commitment to listening and responding is essential to honour the value advisors add to projects.
Evaluating meaningful patient engagement	• Participants expressed desire to use the survey results to identify and prioritize actions to improve patient engagement and measure progress.

Using this evidence, the Working Group outlined recommendations and proposed actions to improve current practice and strengthen patient engagement across AHS (Table 2). These outputs build on existing supports and include opportunities to improve resources, foster inclusivity, and promote collaborative learning opportunities. The recommendations correspond to the overarching categories of evidence-based patient and family engagement indicators described by Santana et al. (19).

**Table 2:** Recommendations and actions to optimize patient and family engagement across Alberta's learning health system.

Recommendation	Proposed actions	Patient engagement indicator category
1. Increase awareness of existing knowledge and support resources (i.e., *Engaging for Excellence* guides, PERG, staff liaisons, orientations) and allocate resources (staff and funding) to support engagement activities.	• Update resources to offer a standard for patient engagement • Create a communication and learning strategy • Assign a designated contact person • Have more than one advisor involved in projects	Supports for engagement
2. Design a mentorship program for staff and advisors.	• Co-build a mentorship model • Launch and evaluate a mentorship program	
3. Develop a shared understanding to guide patient engagement within research initiatives, and extend training on engagement methods, facilitation and consensus-building to advisors and research communities.	• Host collaborative learning sessions (e.g., lunch and learns) • Share topic-specific resources • Continue collaboration with the Alberta Support for Patient-Oriented Research Unit (AbSPORU)	Working together
4. Ensure that engagement activities represent the diversity of Albertans.	• Build relationships across the population • Use appropriate forms of recognition and appreciation • Use diverse engagement resources and methods (e.g., taking initiatives to community settings, partnering with established groups, and utilizing rural meeting spaces)	Diversity of perspectives
5. Evaluate patient engagement activities by repeating the Patient Engagement Indicator Survey every two years.	• Extend future surveys to reach a varied group of participants • Conduct shorter versions of the survey to monitor progress • Occasional check-ins to reinforce continuous patient engagement	Evaluating meaningful patient engagement

Implementing these recommendations will require involvement of multiple partners, with action planning co-led by patient advisors and the AHS Engagement and Patient Experience team. Potential barriers to implementation include workforce capacity and resource constraints (financial and personnel to champion these initiatives), particularly amid other challenges and priorities facing health delivery providers.

Within Alberta, significant changes are underway across the health system, including creation of new provincial health agencies and supporting structures. As these transitions proceed, we anticipate that patient engagement will remain an integral part of Alberta's LHS. Future research directions may include studying staff experiences and needs regarding patient engagement; however, practical considerations (e.g., capacity constraints and access to frontline staff) may pose barriers to further study or implementation at this time.

## Discussion

Patient advisors have an important role to play in optimizing processes around engagement and aligning practices with best principles for co-design and patient-centred care. The evidence-based, co-designed PFE-Is and survey presented here are ready for implementation as standardized tools to monitor and evaluate engagement activities, gather feedback from advisors and staff, and refine existing practices and strategies for patient engagement. The 2023 data provide a baseline from which to measure and report on patient and family engagement, assess performance, and inform actions and decision making.

Alberta's healthcare delivery structures continue to evolve. In 2024, AHS created eight acute care Program Improvement and Integration Networks (PINs), focusing on key health system improvement priorities in cardiovascular health, children's health, critical care, emergency and emergency medical services, medicine, neuroscience and stroke, surgery, and women's health. PINs picked up where SCNs ended and continued to embed patient and family advisors as well as specialists in health informatics, data and analytics, and senior scientists from the Office of Partnerships for Health Services Research, Innovation and Improvement.

Although the voices and inclusion of patient and family advisors have remained within AHS during its transition from a provincial health agency to a hospital service provider, the level of engagement and scope of work appear to have narrowed (at least temporarily) amid extensive organizational change (21). While the advisor role will likely continue to evolve, we anticipate that the Engagement and Patient Experience team, Alberta's provincial programs and refocused health agencies, will continue working with patient and family advisors and other key partners to achieve the goals of an LHS. This approach aligns with theoretical LHS frameworks such as the Reid et al. model, in which patient, caregiver, provider and community co-design is at the centre of the LHS, and patients' and community insights and perspectives are important drivers and “accelerants” for learning, improvement and transformation (3).

## Data Availability

The original contributions presented in the study are included in the article/supplementary material. Further inquiries can be directed to the corresponding author/s.
